# Ultraviolet Irradiation Surface Treatment to Enhance the Bonding Strength of Polyamide-Based Carbon Fiber-Reinforced Thermoplastic Polymers

**DOI:** 10.3390/polym16202864

**Published:** 2024-10-10

**Authors:** Mun Young Hwang, Soon Ho Yoon, Minkook Kim

**Affiliations:** 1 Korea Automotive Technology Institute (KATECH), Cheonan-si 31214, Republic of Korea; myhwang@katech.re.kr; 2 Institute of Advanced Composite Materials, Korea Institute of Science and Technology (KIST), Jeonbuk 55324, Republic of Korea

**Keywords:** surface treatments, adhesion by chemical bonding, thermoplastic composite, UV irradiation

## Abstract

Adhesive bonding is a suitable joining method to satisfy the increasing industrial demand for carbon fiber-reinforced polymers without the need for a machining process. However, thermoplastic-based carbon fiber-reinforced polymers have small adhesive strength with structural thermoset adhesives. In this study, an ultraviolet irradiation surface treatment was developed to improve the adhesive bonding strength for polyamide-based carbon fiber-reinforced polymer. The type of ultraviolet wavelength, irradiation distance and irradiation time were optimized. Surface treatment with simultaneous UV irradiation of 185 nm and 254 nm wavelength generated unbound N-H stretching that was capable of chemical bonding with epoxy adhesives through a photo-scission reaction of the amide bond of polyamide matrix. Therefore, ultraviolet irradiation treatment improved the wettability and functional groups of the polyamide-based carbon fiber-reinforced polymers for adhesive bonding. As a result, the adhesive strength of the polyamide-based carbon fiber-reinforced polymers was increased by more than 230%.

## 1. Introduction

The use of carbon fiber-reinforced polymer composites (CFRPs) in various structures that require weight reduction is increasing because of their excellent mechanical properties. Compared to various fiber-reinforced polymers based on a glass, aramid, and basalt fiber, CFRPs have high specific stiffness and strength that exceed that of steel and aluminum and are attracting attention as the only lightweight material that can replace metal.

CFRPs are categorized into two classes with respect to the type of matrix: the first class is thermosetting polymer matrix-based CFRP, and the second class is thermoplastic polymer matrix-based CFRTPs (carbon fiber-reinforced thermoplastic polymers). CFRPs do not melt upon heating after curing because of the three-dimensional, cross-linked structure in the thermosetting polymer resin. CFRPs have advantages such as easy impregnation and low manufacturing temperature [1]. However, CFRPs have the disadvantages of a long curing time and restricted recycling potential. CFRTPs can be not only remelted when the temperature increases but also rapidly and appropriately molded by extrusion and injection. CFRTPs also have advantages such as excellent impact resistance and fracture resistance, as well as recyclability and extended storage periods [2]. The poor interface between thermoplastic matrix and carbon fiber has been pointed out as a disadvantage; however, it has been recently resolved through varied methods including sizing agents and fabrication processes [3,4]. Therefore, CFRTPs have become more interesting in the market due to their advantages of recyclability and productivity [5].

Joining technology for CFRTPs is important for application to the components of structures. Mechanical joining processes, including riveting and bolting, are appropriate for the assembly of structural metal parts. On the other hand, applying mechanical joining to CFRTPs is difficult because of their low hardness. Moreover, the CFRTP strength is considerably decreased due to delamination and fiber breakage in the preprocessing of hole machining for mechanical joining [6]. Adhesive bonding is a suitable process for combining different parts composed of CFRTPs without the need for a machining method. The adhesive bonding process has several benefits in assembling components, such as decreased weight for structures, acoustic insulation, reduced corrosion, vibration damping, and uniform stress distribution [7,8]. The primary drawback of adhesive bonding lies in its dependency on the nature and state of the surface between the adhesive and the adherend, which dictates the bond strength. The weak polarities, limited reactivity, and low surface energies inherent in thermoset adhesives result in a diminished level of adhesion with the thermoplastic matrix [9].

To address these constraints, various surface treatments have been developed to enhance the adhesion strength of CFRTPs. The main objectives of surface treatments are to eliminate contaminants, increase the surface free energy and roughness, and enhance adhesion by activating the adhesion layer [10]. Mechanical surface treatment of CFRTPs, such as grit (sand) blasting, grinding, sanding, and shot peening, can occasionally damage the carbon fiber reinforcement, resulting in the degradation of mechanical properties and delamination of the CFRTP itself [11,12]. Chemical surface treatment methods, such as acid oxidation and coupling agent treatments, have been utilized to enhance bonding between CFRTP and adhesives without mechanical damage [13,14,15].

Physical surface treatment methods using plasma, laser, and ultraviolet light (UV) are also widely researched [16,17]. Low-pressure plasma treatment is one of the most effective surface treatments for enhancing the bonding strength of CFRTP. This treatment has advantages such as contaminant removal, surface activation, functional group induction, and no need for hazardous chemicals [18]. However, plasma treatment requires an expensive and time-consuming vacuum system because atmospheric plasma treatment is relatively ineffective [19,20]. Laser radiation treatment has the potential for efficient automation and short processing time and provides non-contact interaction [21]. Since the laser beam size is less than 1 mm, precise processing is possible, but it is difficult to apply to a large area. Laser radiation can cause thermal damage to the substrate, which is particularly fatal for FRPs [22].

UV irradiation surface treatment can be applied to a large area at an affordable cost and minimizes surface damage. UV irradiation has been primarily initiated to eliminate organic pollution on the surfaces of different polymers. Numerous studies have shown that using UV irradiation surface treatment on polymer surfaces can degrade the matrix and produce oxidation, cross-linking and chain scission [23,24,25]. Consequently, UV irradiation is often utilized as another surface treatment for enhancing the wettability of thermoplastic polymer [26]. Mathieson et al. [24] selected UV irradiation treatment to activate the surfaces of thermoplastics such as polyether ether ketone (PEEK) and polyethylene (PE) plastics in the adhesive bonding process. Shi et al. [27] used the PEEK films with UV irradiation treatment as adhesives to bond thermosetting composites by a co-curing process. Quan et al. [28] improved the bonding strength of polyphenylene sulfide (PPS) and PEEK composites by irradiation with high-energy UV for a short time. The results reported in the literature clearly show the excellent effect of UV irradiation treatment for improving the adhesion between thermoplastic adherends and thermoset adhesives.

However, minimal attention has been given to the mechanical damage generated in CFRTPs by the use of high-energy UV irradiation for adhesive bonding. An excessively high-energy treatment induces excessive deterioration of the CFRTP surface (damage to the matrix) and can cause a decline in adhesion quality. Therefore, it is desirable to identify the optimal process conditions for UV irradiation to significantly improve the adhesive strength and to minimize damage to the materials.

In this study, the effect of UV irradiation treatment on CFRTP was investigated to enhance the adhesive bonding strength. The adhesive shear bonding strength of the polyamide6-based CFRTP was analyzed with respect to the mechanism of adhesion; it was evaluated and optimized by using the single-lap shear adhesive test with ultraviolet C (UVC), irradiation time and distance. Failure mode analysis, surface morphology, measurement of surface free energy, and FTIR analysis were also employed to investigate the mechanisms for enhancing the bonding strength. The specific type of UVC had to significantly improve the chemical interaction and wettability of the polyamide-based CFRTPs to the epoxy adhesive. The optimal conditions of UV irradiation treatment were determined.

## 2. Experimental

### 2.1. Materials and Fabrication

The CFRTP was composed of carbon fiber/polyamide 6 prepregs (Kolon, Gyeonggi-do, Republic of Korea). CFRTP adherend specimens were fabricated to 3 mm with cross ply stacking sequence [0_2_/90]_3S_ using an autoclave process. The process temperature and pressure were maintained at 250 °C and 7 bar for 30 min. The heating and cooling rates of the autoclave process cycle were 4 °C/min. The density, fiber volume fraction, and void volume fraction of the CFRTP was 1.4 g/cm^3^, 57.0%, and 1.2%, respectively, after the fabrication. Tensile properties and interlaminar shear strength (ILSS) of CFRTP were measured following ASTM D3039 [29] and ASTM D2344, respectively. Table 1 shows the material properties of the polyamide 6 (PA6)-based CFRTP adherend.

### 2.2. UV Irradiation Surface Treatment

The CFRTP surface was treated using medium-wavelength ultraviolet (UVB, wavelengths from 280 to 315 nm) and short-wavelength ultraviolet (UVC, wavelengths from 100 to 280 nm) lamps of spectral wavelengths. In this study, three types of lamps were employed: (1) lamps that emit at a wavelength of 312 nm UVB, (2) lamps that emit only at a wavelength of 254 nm UVC (UVC^254^), and (3) lamps that simultaneously emit at wavelengths of 185 nm and 254 nm UVC (UVC^185+254^). The CFRTP adherends were cleaned with acetone and dried at 60 °C in a dry oven prior to treatment. During the treatment, the CFRTP adherends were placed in a chamber (320 mm × 300 mm × 120 mm) with six 20 W tube UVB or UVC lamps with a diameter of 16 mm positioned 10 mm apart. The irradiation distances between the lamps and the CFRTP surface were controlled to be 30 mm, 15 mm, and 5 mm. The irradiation times were controlled to be 5 min, 15 min, 30 min, 60 min, 90 min, 120 min, and 300 min to optimize the treatment conditions for the maximum adhesive bonding strength. The UVB and UVC irradiation treatments were carried out in laboratory air at a temperature of 23 °C and a relative humidity of 50%. The lamps were preheated for 5 min before irradiation.

The UVC^185+254^ irradiation treatments were performed with different UVC^185+254^ dose energies. The UVC^185+254^ dose energy was calculated by Equation (1). Table 2 indicates UVC^185+254^ dose energy for irradiation distances of 5 mm and 15 mm:

$$\mathrm{UV}\;\mathrm{dose}\;\mathrm{energy}\;\left[J/{\mathrm{cm}}^{2}\right]=\mathrm{UV}\;\mathrm{intensity}\;\left[W/{\mathrm{cm}}^{2}\right]\times \mathrm{irradiation}\;\mathrm{time}\;\left[s\right]$$

(1) UV intensity ∝ 1UV irradiation distance2

where the UVC^185+254^ intensity of the lamp was 3 mW/cm^2^ per lamp measured at a distance of 25 mm.

### 2.3. Single-Lap Shear Test

An epoxy adhesive film (Epoxy sheet 54, Singsung composite materials, Seoul, Republic of Korea) was selected for adhesive bonding. A thickness gauge of polytetrafluoroethylene (PTFE) tape (Air-tech, Huntington Beach, CA, USA) was selected to control the adhesive layer thickness and area (15 × 15 × 0.32 mm^3^). The epoxy file adhesives were cured for 2 h by the vacuum bag process for degassing using hot oven at 120 °C. Table 3 shows the material properties of the epoxy adhesive film obtained by the manufacturer.

**Figure 1:**
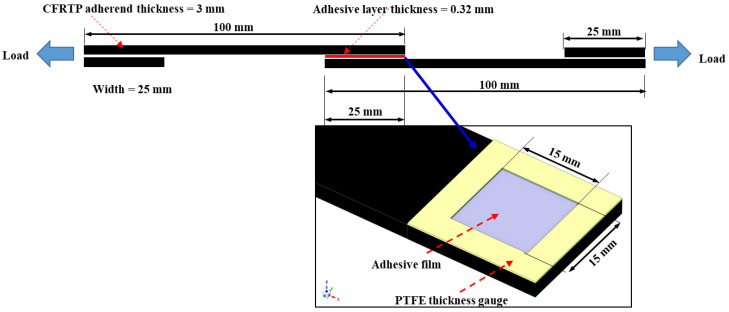
Schematic of the single-lap shear test specimen configuration.

To examine the bonding strength between the CFRTP and the epoxy adhesive, the single-lap shear strength was measured according to the ASTM D3163-01 standard. The bonding shear strength is an important property for planning the joining of structural components and for understanding the stress distribution in the adhesive layer. Specimens were performed immediately after the treatment to minimize the decrease in the UV effect over time. To decrease the eccentricity of the load path and nonuniform shear stress during the test, 25 × 25 mm^2^ tabs were attached on both ends as shown in Figure 1. All tests were repeated 5 times using a universal test machine (Instron 5985, Instron, Norwood, MA, USA) with a crosshead speed of 2 mm/min.

### 2.4. Failure Mode and Wettability in Specimens

The morphology of CFRTP and the fractured adhesive surface was observed and analyzed by using a digital microscope (VHX-900F, Keyence, Osaka, Japan). The CFRTP surface free energies at different irradiation times were calculated based on contact angle measurement results. The irradiation distance was fixed at 5 mm. The interaction of molecular adsorption is based on the polar and dispersive fraction of the surface free energy. The molecular interaction can determine the bonding strength of the interfacial layer. The solid surface free energy can be calculated according to the Owens–Wendt–Rabel–Kaelble (OWRK) model using the contact angle with two known liquids, as shown in Equation (2) [30]:

$$\gamma_{lv}\left(1+cos\theta \right)=2\sqrt{\gamma_{sv}^{d}\gamma_{lv}^{d}}+2\sqrt{\gamma_{sv}^{p}\gamma_{lv}^{p}}$$

(2) $$\mathrm{Surface}\;\mathrm{free}\;\mathrm{energy}\;\left(\gamma_{sv}\right)=\gamma_{sv}^{d}+\gamma_{sv}^{p}$$

where θ is the measured contact angle, $\gamma_{lv}$ is the surface tension, $\gamma_{lv}^{d}$ and $\gamma_{lv}^{p}$ are the dispersive and the polar component surface free energy of the liquid, $\gamma_{sv}^{d}$ and $\gamma_{sv}^{p}$ are the dispersive and the polar component surface free energy of the solid.

The total sum of surface free energy $\gamma_{sv}$ of the adherend is the total of the dispersive $\gamma_{sv}^{d}$ and polar $\gamma_{sv}^{p}$ components. The two separated unknown variables in Equation (2), $\gamma_{sv}^{d}$ and $\gamma_{sv}^{p}$, and the solid surface free energy can be determined by observing the contact angle with two different liquids. The contact angles and surface free energy were measured using distilled water and glycerol, and all measurements were repeated five times. Table 4 indicates the parameters of the surface tension in distilled water and glycerol liquid.

### 2.5. Fourier Transform Infrared Spectroscopy (FTIR)

FTIR analyses of the CFRTP surfaces were conducted to estimate the surface functional groups of the CFRTP surface according to the UVC irradiation surface treatment. FTIR was performed by an attenuated total reflectance device (6600FV, Jasco, Tokyo, Japan) with a diamond crystal. The spectral range, resolution, and scan points of the measurement was 750 cm^−1^ to 4000 cm^−1^, 4 cm^−1^, and 32 points, respectively.

## 3. Results and Discussion

### 3.1. Analysis of Surface Morphology

**Figure 2:**
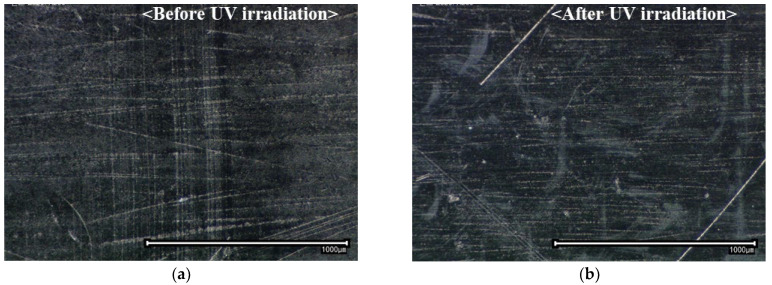
Surface morphology of the CFRTP adherend: (**a**) before and (**b**) after UVC^185+254^ treatment for 300 min with an irradiation distance of 5 mm.

Figure 2 indicates the surface morphology of the CFRTP before and after UVC^185+254^ irradiation treatment for 300 min with an irradiation distance of 5 mm. Different from mechanical surface treatment such as sandblasting or sanding, there were no significant roughness changes or cracks to the surface morphology due to UVC^185+254^ irradiation. This finding proves that the UVC irradiation treatment did not cause critical damage that degraded the mechanical properties (fiber breakage, cracks on the surface matrix or delamination between the fiber and the matrix) to the surface of the PA6 matrix CFRTP within the UVC^185+254^ irradiation time. The relatively low energy of UVC irradiation compared with other energy-based surface treatment methods was the main factor [18].

### 3.2. Adhesive Shear Strength

**Figure 3:**
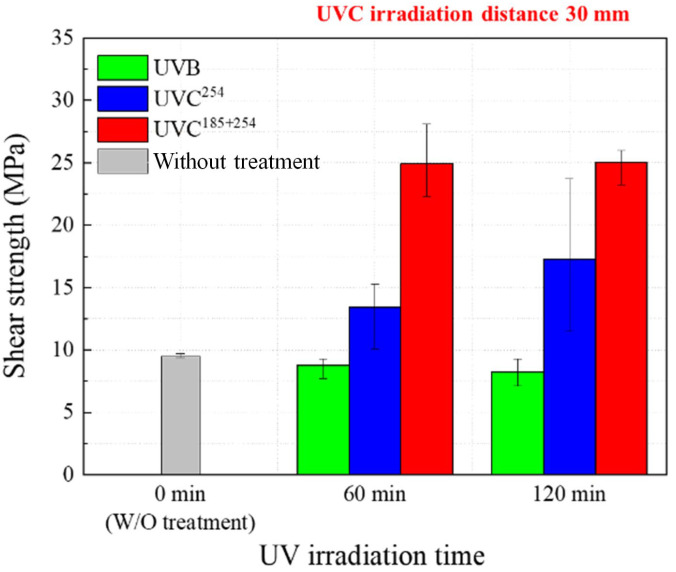
Shear bonding strength with respect to the UV type and irradiation time.

Figure 3 shows the shear strength with respect to the UV type and irradiation time. The UV irradiation distance was 30 mm. The joint shear strength of untreated CFRTPs without surface treatment was 9.5 MPa, which is a relatively low strength. The UVB irradiation treatment had no effect on the joint shear strength of the CFRTPs. In the specimens treated by UVB, the maximum joint shear strength was 9.3 MPa, which is equivalent to those of the cases without surface treatment. Although the UVB irradiation time increased to 120 min, there was no improvement in the joint strength. For the case of UVC^254^, the joint shear strength increased to 13.4 MPa and 17.3 MPa with irradiation times of 60 min and 120 min, respectively. The UVC^185+254^ irradiation treatment showed the most effective improvement for the joint strength. The joint shear strength of CFRTPs increased to 24.9 MPa and 25.1 MPa with 60 min and 120 min, respectively, of UVC^185+254^ irradiation treatments. The joint shear strength improvement effect of the UVC^185+254^ irradiation treatment was more than twice that of UVC^254^.

**Figure 4:**
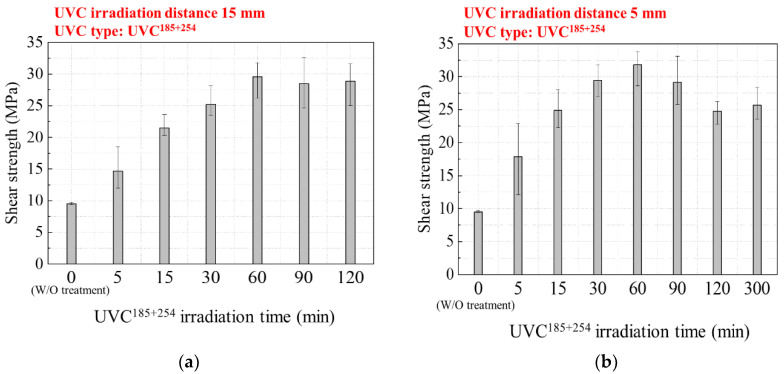
Shear bonding strength according to UVC^185+254^ irradiation time and distance: (**a**) 15 mm and (**b**) 5 mm.

The irradiation treatment time and distance of the UVC^185+254^ irradiation treatments, which showed a significant enhancement, were optimized. Figure 4 shows the joint shear strength of the CFRTPs with respect to the UVC^185+254^ irradiation time and distance. For UVC^185+254^ irradiation distances of 15 mm, the shear strengths were maximized with an irradiation time of 60 min. The joint strength increased by 210% to 29.6 MPa for an irradiation time of 60 min.

Similarly, for an irradiation distance of 5 mm, the shear strength increased by 234% to 31.8 MPa by the 60 min irradiation time. With the irradiation time of 30 min and 90 min with 5 mm distance, the joint shear strength was 29.5 MPa and 29.1 MPa, respectively. To statistically analyze the difference between joint shear strength results of the 30, 60, and 90 min irradiation time, a *t*-test was performed under the null hypothesis. There was no statistical difference as a result of the *t*-test with a significance level of 0.05. It means that the joint shear strength of the CFRTPs was maximized up to 30 MPa by the UVC^185+254^ treatment for more than 30 min irradiation time at a 5 mm irradiation distance.

The interlaminar shear strength (ILSS) of the carbon/PA6 CFRTP is approximately 30 MPa [31]. Therefore, the limit of the joint shear strength of the CFRTPs is 30 MPa because shear failure (delamination) of the CFRTP adherend will occur even if adhesive or cohesive failure does not occur. When the UVC^185+254^ irradiation time was increased above 30 min, the shear strength either became maximized to 30 MPa. In particular, for the case of an irradiation distance of 5 mm, the joint shear strength decreased to 25 MPa with an irradiation time of 120 min. This result is attributed to a decrease in the intrinsic CFRTP adherends strength by deterioration of the PA6 matrix upon UV exposure [32].

### 3.3. Wettability and Surface Free Energy Analysis

**Figure 5:**
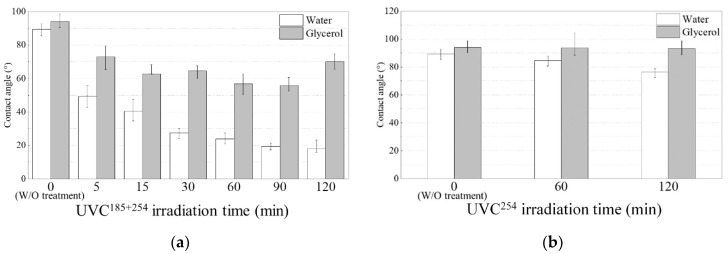
Distilled water and glycerol contact angles on the CFRTP surface with respect to the irradiation treatment time: (**a**) UVC^185+254^ and (**b**) UVC^254^.

The wettability of the solid surface and liquid is dominant to the surface free energy. The distilled water and glycerol liquid contact angles on the CFRTP are shown in Figure 5, according to the irradiation treatment time at an irradiation distance of 5 mm, which is the same condition as in Figure 4b. All measurements were conducted within 1 h after UVC surface treatment. The untreated CFRTP surface without UVC irradiation showed contact angles of 89° and 93° with distilled water and glycerol, respectively. The water contact angle of CFRTPs treated with UVC^185+254^ was continuously reduced in proportion to the irradiation time, as shown in Figure 5a. And the glycerol contact angle was minimized to 55° by the 60 min and 90 min irradiation times. However, in case of the UVC^254^, the glycerol contact angle was not changed, and the water contact angle was only reduced to 75°, as shown in Figure 5b. This shows that the joint shear strength improvement from the UVC^254^ irradiation treatment is attributed to the removal of contamination on the surface.

The water contact angle was determined by the polar component of the surface energy, and the glycerol contact angle was determined by the dispersive component. The dispersive component specifically denotes the van der Waals interaction, while the polar components encompass various interactions between dipoles, including Coulomb interactions and hydrogen bonding [30]. Table 5 shows the surface free energy of the CFRTPs according to both UVC irradiation times with a 5 mm irradiation distance.

Similar to the results in Figure 4, the surface free energy was greatly increased up to 107 mJ/m^2^ after 30 min of UVC^185+254^ irradiation and then became saturated. On the other hand, the UVC^254^ irradiation treatment produced CFRTP surface free energy with a slightly increased polar component, which led to the higher enhancement of the adhesive strength by UVC^185+254^ compared with that of the UVC^254^ treatment, as shown in Table 5. It is obvious that UVB light has lower energy than UVC254; therefore, it has no effect on surface free energy.

As the irradiation time of UVC^185+254^ was increased up to 30 min, the dramatic increases in the joint shear strength of the CFRTP were attributed to the increase in surface free energy. When the UVC^185+254^ irradiation time was 120 min, the surface energy increased to 132 mJ/m^2^; however, the adhesion shear strength decreased. This reduction is due to damage to the surface matrix layer, and surface FTIR analysis was performed to demonstrate this.

### 3.4. Analysis of FTIR Results

**Figure 6:**
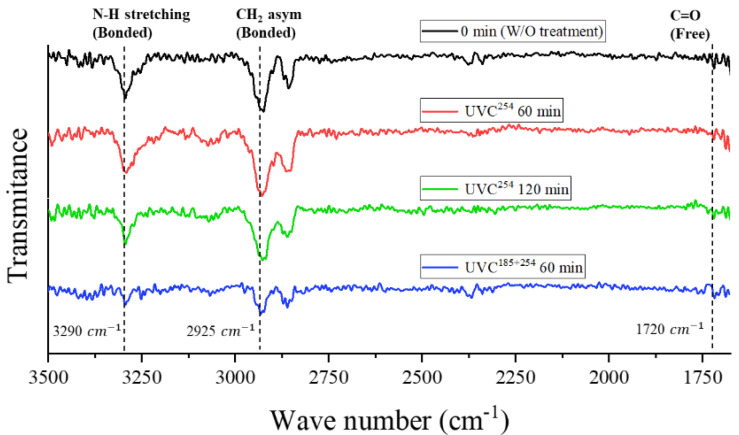
FTIR spectra of the CFRTP surface according to the UVC types.

The ATR-FTIR method was employed to examine the surface of UVC-treated CFRTPs, aiming to analyze both functional groups and chemical alterations. Figure 6 shows the CFRTP surface FTIR spectra with different UVC irradiation treatment conditions. The peak at 3290 cm^−1^ corresponds to the N–H stretching vibration 38, and the peak at 2925 cm^−1^ corresponds to asymmetric –CH_2_– stretching vibrations. As shown in Figure 6, there are no changes in the peaks at 3290 cm^−1^ and 2925 cm^−1^ with UVC^254^ irradiation. For the case of UVC^185+254^ irradiation, in contrast, the peak intensities at 3290 cm^−1^ and 2925 cm^−1^ are markedly decreased, which means that UVC^185+254^ irradiation treatment chemically modifies the surface of CFRTPs. The energy (E) of UV light is given by Equation (3) [33]:

(3) $$E=\mathrm{Nhc}/\lambda \times 105\left(\mathrm{kJ}/\mathrm{mol}\right)$$

where N, h, c and λ denote Avogadro’s number, Planck’s constant, light speed, and wavelength, respectively. The energies of 254 nm and 185 nm wavelength UVC are 472 kJ/mol and 645 kJ/mol, respectively, which are higher than the bonding energies of single chemical bonds of the polyamide: C–C, C–H, C–O, and C–N are 347.7 kJ/mol, 413.4 kJ/mol, 351.5 kJ/mol, and 260 kJ/mol, respectively [34]. However, UVC^254^ does not have enough potential energy to debond the chemical bonding of the polyamide, although the 254 nm wavelength UVC has higher energy than that of single chemical bonds.

The amide bonds of the PA6 matrix were decomposed with the photo-scission reactions by UVC^185+254^ [32], which can be confirmed by the decreased peak intensities of the N–H and –CH_2_– stretching vibrations, as shown in Figure 6. The photo-scission process results in the vaporization of the carbonyl group (C=O), leading to the generation of carbon monoxide (C≡O) and an elevation in the presence of unbound N–H (without hydrogen bond) and unbound –CH_2_– stretching on the surface of the CFRTP [35]. The unbound N–H stretching establishes hydrogen bonds with the –OH group of the epoxy adhesive, enhancing interfacial bonding through radicals [36]. However, the N-H stretching initially present in PA6 is already engaged in hydrogen bonding with the C=O in PA6 and is unable to make a hydrogen bond within the epoxy [35].

**Figure 7:**
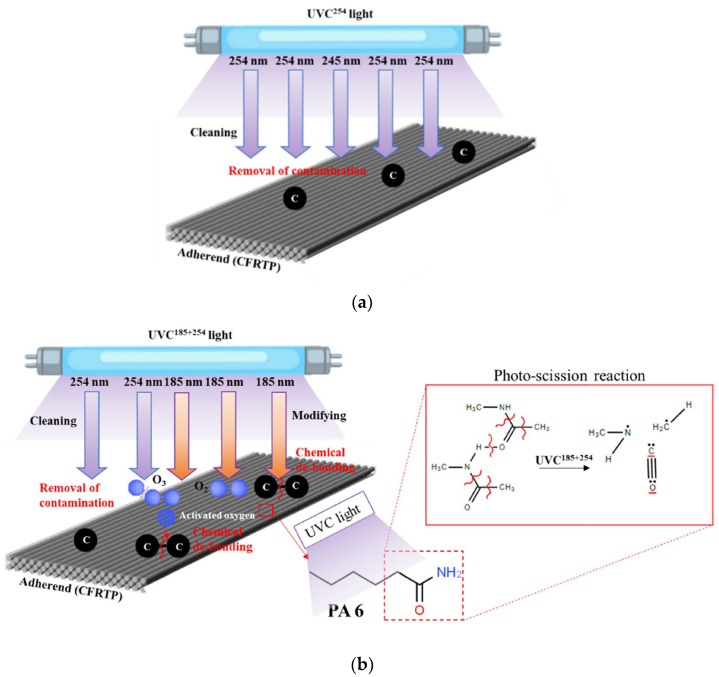
Mechanism for the surface treatment of polyamide-based CFRTP: (**a**) UVC^254^ irradiation and (**b**) UVC^185+254^ irradiation.

The surface modification of PA6 by UVC begins by eliminating organic contamination from the surface. The mechanism of UVC light cleaning and chemical modification is chiefly based on two wavelengths, 185 nm and 254 nm, as shown in Figure 7. UVC with wavelength lines at 185 nm generates ozone with O_3_ and atomic oxygen with O by photolysis of oxygen gas in an endothermic reaction. In contrast, UVC with wavelength lines at 254 nm is easily absorbed by the single C–C bonds of hydrocarbons and ozone. The UVC chemical modification process is a continuous oxidation process started at reactive oxygen (and carbon) radicals [37,38]. Of course, no surface cleaning (remove the contaminate) can be expected from low-energy UVB surface treatment, and therefore, it has no effect on improving the adhesion-bonding strength of the CFRTP.

**Figure 8:**
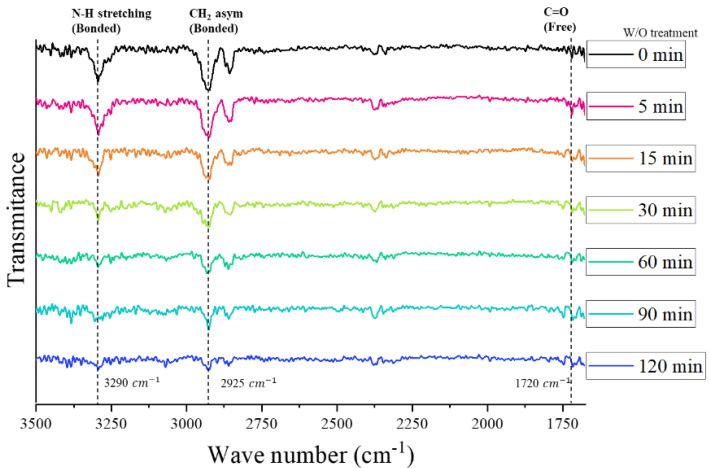
Transmittance spectra of the PA6 matrix CFRTP surface according to the UVC^185+254^ treatment time.

At the beginning of the treatment (before 15 min), the N–H and –CH_2_– stretching vibration peaks did not significantly change, and after 30 min of irradiation, the N-H and –CH_2_– stretching vibration peaks were rapidly reduced, as shown in Figure 8. The chemical modification (photo-scission reaction of the amide bonds) occurred after irradiation with UVC^185+254^ for 30 min. The joint shear strength was increased by this chemical modification and maximized with a 30 min irradiation time. Even after attaining the maximum value of joint shear strength, the PA6 matrix of the CFRTPs continued to degrade via the photo-scission reaction of UVC^185+254,^ and the intensity levels at 3290 cm^−1^ and 2925 cm^−1^ steadily decreased. Those two intensity levels were significantly decreased with 120 min irradiation time, as shown in Figure 8. The continued decomposition of the PA6 matrix on the surface deteriorates the mechanical properties of the CFRTP itself [39]. The damaged CFRTP surfaces are easily delaminated or fail, and consequently, the joint shear strength is also reduced when the irradiation time is longer than 120 min.

### 3.5. Failure Mode Analysis

**Figure 9:**
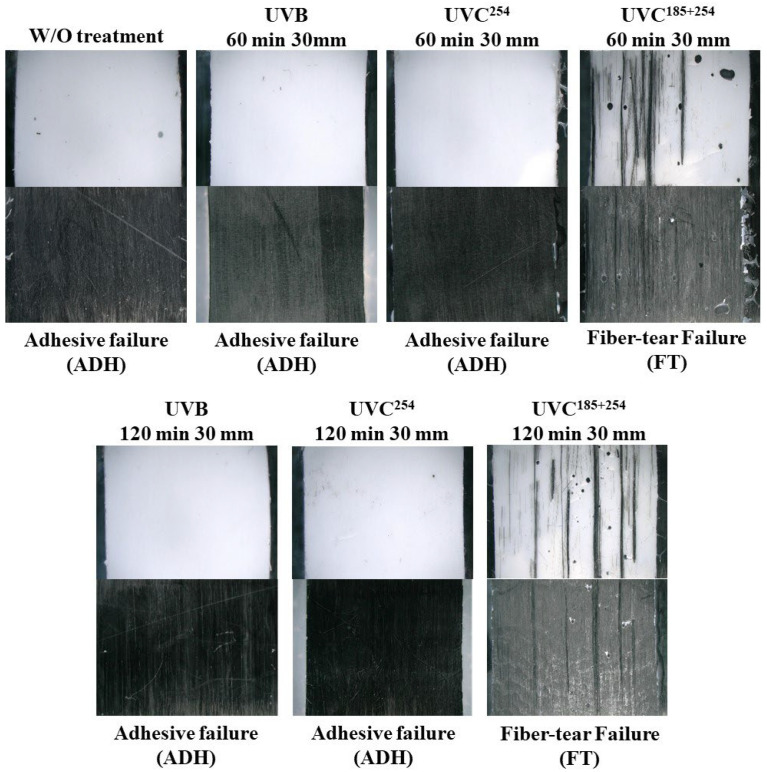
Fracture surfaces and failure modes of the adhesive joint with respect to the UV irradiation condition.

The interface of an adhesive layer on a CFRP is composed with the matrix surface not directly bonding with fiber reinforcement. The failure modes of the adhesive bonding are categorized as cohesive failure, interface failure, fiber-tear (substrate) failure, and light tear failure [40]. Figure 9 shows the fracture surface images of the single-lap shear adhesive joint with respect to the type of UV treatment. In the absence of UV irradiation treatment, adhesive failure (ADH) occurred as a result of a weak interface between the epoxy adhesive layer and pure CFRTP surface. ADH was the dominant failure mode even for the UVB- and UVC^254^-treated CFRTP specimens. However, the fiber-tear failure (FT) mode was observed for the case of CFRTPs treated with UVC^185+254^. The interfacial bonding strength between the epoxy adhesive and the CFRTP adherend was obviously enhanced by the UVC^185+254^ irradiation. UVB and UVC^254^ irradiation cannot sufficiently modify the surface to enhance the interfacial bonding between the adhesive and the CFRTP surface.

Compared to the untreated case, the deviation of the bonding shear strength with UV irradiation treatment was significantly large. The basic mechanism for improving adhesion is due to a chemical reaction; there is a difference in the surface treatment effect even under the same conditions. In addition, as surface treatment progresses, deviation of the adhesion strength tends to increase further because of complex failure modes such as fiber tear and cohesive failure.

**Figure 10:**
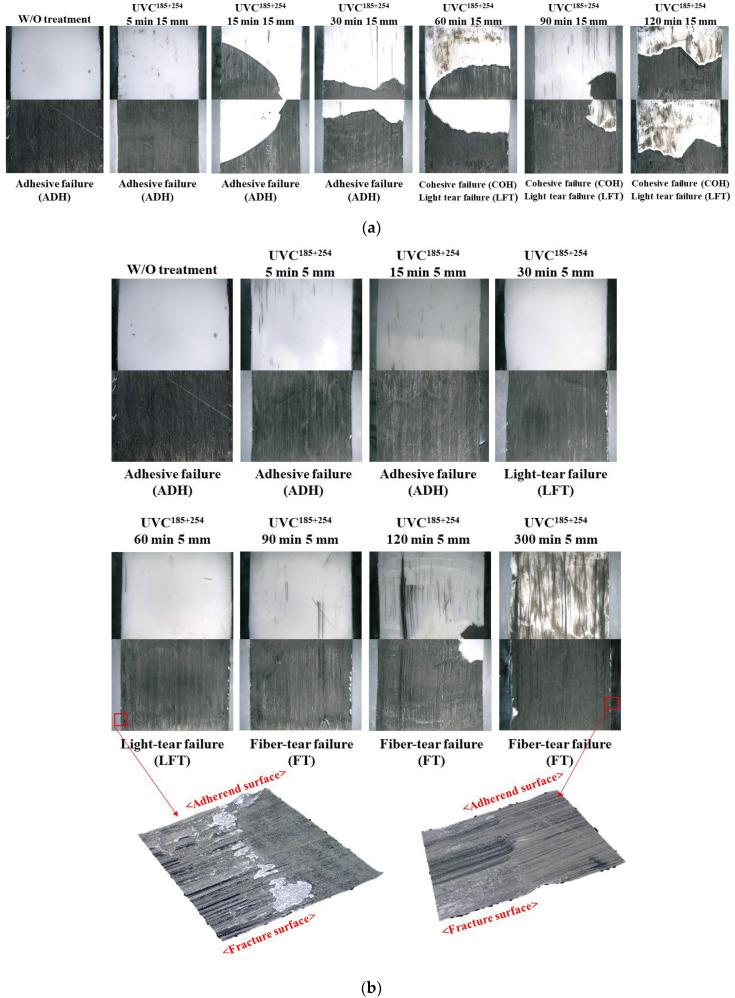
Fracture surfaces and failure modes of an adhesive joint according to the UVC^185+254^ irradiation time. Irradiation distance: (**a**) 15 mm and (**b**) 5 mm.

Figure 10 shows the fracture surface images of the single-lap shear adhesive joint with respect to the UVC^185+254^ irradiation time and distance. The failure behavior of the CFRTP joints transformed from ADH to light tear failure (LFT) with increasing shear adhesive strength. In the case of the 15 mm irradiation distance, the failure mode was ADH until a 30 min irradiation time was reached, suggesting a relatively low interfacial interaction between the adhesive and substrate and the bonding strength, as shown in Figure 10a. Next, the failure mode changed to LFT by the enhanced interfacial bonding due to the photo-scission reaction. When the irradiation time exceeded 60 min, LFT was sustained, corresponding to the saturated shear adhesive strength.

With a UVC^185+254^ irradiation distance of 5 mm, the failure behavior changed to LFT after 30 min and 60 min because of the exposure to higher energy at shorter irradiation distances. The optimum joint shear strength was obtained because the CFRTP did not deteriorate, and only the PA6 matrix on the surface was photo-scissed by UVC^185+254^ irradiation to form strong interfacial bonding with the epoxy adhesive [31]. For an irradiation time longer than 90 min, the failure mode started to change to FT because the matrix deteriorated by prolonged UV exposure, decreasing the intrinsic strength of the CFRTP substrate. In particular, with 300 min of irradiation, a thin layer of the carbon fiber was delaminated on the surface of the CFRTP, as shown in Figure 10b. Thus, the ILSS of the CFRTP surface was weaker than the adhesive bonding strength with the epoxy adhesive. As a result, considering the treatment condition of the maximum joint shear strength, 30 MPa is the optimum condition for increasing the interfacial bonding by chemical modification of the matrix with minimum damage to the CFRTP substrate.

## 4. Conclusions

In this study, the effect of ultraviolet irradiation treatment was investigated and optimized to enhance the adhesive strength of polyamide-based CFRTPs. It was determined that the joint shear strength of single-lap specimens was significantly increased by UVC^185+254^ irradiation treatment; on the other hand, UVC^254^ and UVB exhibited no significant improvement. The joint shear strength of the CFRTP increased by 230% to 30 MPa by the UVC^185+254^ irradiation treatment for 30 min at a distance of 5 mm.

The surface free energy, FTIR, and failure mode were analyzed to understand the mechanism of the increased joint shear strength. At the beginning of the UVC^185+254^ irradiation treatment, the adhesive strength is increased by the surface energy improvement due to the removal of organic contaminants. For the subsequent treatment, chemical modification occurs on the surface of the PA6 matrix by UVC^185+254^ irradiation. The photo-scission reaction of the amide bond of the polyamide produces unbound N-H stretching, which then establishes hydrogen bonds with the O-H group of the epoxy adhesive. The unbound N-H stretching generated by the UVC^185+254^ irradiation treatment forms strong hydrogen bonds with the epoxy adhesive; as a result, the adhesive bonding shear strength of the PA6-based CFRTP with the epoxy adhesive increases to higher than the ILSS of the CFRTP. However, the PA6 matrix continues to degrade due to UVC^185+254^ irradiation, and consequently, the adhesive strength decreases after the optimal treatment time due to the deterioration in the intrinsic strength of the CFRTP substrate.

## Figures and Tables

**Table 1:** Material properties of the PA6-based CFRTP adherend.

	Tensile Modulus	Tensile Strength	Poisson’s Ratio	ILSS	Melting Point
PA6 based CFRTP	120 GPa	1800 MPa	0.31	30 MPa	220 °C

**Table 2:** Total UVC^185+254^ dose energy according to the UVC^185+254^ irradiation conditions.

	Irradiation Distance	Irradiation Time (min)						
		5	15	30	60	90	120	300
Total $\mathrm{UV}\;\mathrm{dose}\;\mathrm{energy}\;(J/{\mathrm{cm}}^{2})$	5 mm	72	216	432	864	1296	1728	4320
	15 mm	18	54	108	216	324	432	1080

**Table 3:** Mechanical and thermal properties of the epoxy adhesive.

	Lap Shear Strength with Aluminum	Curing Temperature	Glass Transition Temperature
Epoxy film adhesive	40 MPa	120 °C	78 °C

**Table 4:** Surface tension parameters for distilled water and glycerol (mJ/m^2^).

Material	$\mathrm{Total}\;\mathrm{Surface}\;\mathrm{Tension}\;(\gamma_{lv})$	$\mathrm{Dispersive}\;\mathrm{Component}\;(\gamma_{lv}^{d})$	$\mathrm{Polar}\;\mathrm{Component}\;(\gamma_{lv}^{p})$
Distilled water	72.8	21.8	51
Glycerol	64	34	30

**Table 5:** Surface free energy of the PA6 matrix CFRTPs with respect to the UVC irradiation times.

UVC^185+254^							
Irradiation time (min)	0	5	15	30	60	90	120
$\gamma_{sv}^{d}$ (mJ/m^2^)	0.40	0.30	0.16	0.92	0.12	0.14	3.9
$\gamma_{sv}^{p}$ (mJ/m^2^)	22.6	78.6	87.1	106.6	100.9	104.6	128.4
$\gamma_{sv}$ (mJ/m^2^)	23.0	78.9	87.3	107.5	101.0	104.7	132.3
**UVC^245^**							
Irradiation time (min)	0		60			120	
$\gamma_{sv}^{d}$ (mJ/m^2^)	0.4		0.02			0.35	
$\gamma_{sv}^{p}$ (mJ/m^2^)	22.6		30.3			45.4	
$\gamma_{sv}$ (mJ/m^2^)	23.0		30.4			45.7	

## Data Availability

Data are contained within the article.
